# Structured self-management education maintained over two years in insufficiently controlled type 2 diabetes patients: the ERMIES randomised trial in Reunion Island

**DOI:** 10.1186/1475-2840-11-91

**Published:** 2012-08-02

**Authors:** Xavier Debussche, Fidéline Collin, Adrian Fianu, Maryvette Balcou-Debussche, Isabelle Fouet-Rosiers, Michèle Koleck, François Favier

**Affiliations:** 1 Metabolic and Chronic Diseases Department, Endocrinology, Diabetology and Nutrition Unit, CHU of La Reunion, Saint-Denis, La Reunion, France; 2 Centre for Clinical Investigation-Clinical Epidemiology (CIC-EC INSERM/CHU/University), CHU of La Réunion, Saint-Pierre/Saint-Denis, La Reunion, France; 3 Clinical Research and Innovation Department, CHU of La Reunion, La Reunion, France; 4 LCF (Languages, texts and communication in Creole and French speaking spaces), CNRS- UMR 8143, IUFM-University of La Reunion, Saint-Denis, La Reunion, France; 5 UNiRès, UNiversities/IUFM network for health education, Blaise Pascal University, Clermont-Ferrand II, Chamallières, France; 6 Health Psychology laboratory, EA 487, Victor Segalen University (Bordeaux 2), Bordeaux, France; 7 Diabétologie- Endocrinologie, Hôpital Félix Guyon, CHU de la Réunion, 97405 Saint-Denis, Cedex, France

**Keywords:** Randomised controlled trial, Self-management education, Type 2 diabetes, Learning nests, Reunion Island

## Abstract

**Background:**

Self-management education programs can reduce the complications and mortality in type 2 diabetes. The need to structure these programs for outpatient and community care with a vision for long-term maintenance has been recognised. In Reunion Island, an area affected by epidemiological and nutritional transition, diabetes affects 18% of the adult population over 30 years, with major social disparities, poor glycaemic control and frequent cardiovascular complications.

**Methods/Design:**

ERMIES is a randomised controlled trial designed to test the efficacy of a long-term (2 years) structured group self management educational intervention in improving blood glucose in non-recent, insufficiently controlled diabetes. After an initial structured educational cycle carried out blind for the intervention arm, patients will be randomised in two parallel group arms of 120 subjects: structured on-going group with educational intervention maintained over two years, versus only initial education. Education sessions are organised through a regional diabetes management network, and performed by trained registered nurses at close quarters. The educational approach is theoretically based (socio-constructivism, social contextualisation, empowerment, action planning) and reproducible, thanks to curricula and handouts for educators and learners. The subjects will be recruited from five hospital outpatient settings all over Reunion Island. The main eligibility criteria include: age ≥18 years, type 2 diabetes treated for more than one year, HbA1c ≥ 7.5% for ≥3 months, without any severe evolving complication (ischaemic or proliferative retinopathy, severe renal insufficiency, coronaropathy or evolving foot lesion), and absence of any major physical or cognitive handicap. The primary outcome measure is HbA1c evolution between inclusion and 2 years. The secondary outcome measures include anthropometric indicators, blood pressure, lipids, antidiabetic medications, level of physical activity, food ingestion, quality of life, social support, anxiety, depression levels and self-efficacy. An associated nested qualitative study will be conducted with 30 to 40 subjects in order to analyse the learning and adaptation processes during the education cycles, and throughout the study.

**Conclusions:**

This research will help to address the necessary but difficult issue of structuring therapeutic education in type 2 diabetes based on: efficacy and potential interest of organising on-going empowerment group–sessions, at close quarters, over the long term, in a heterogeneous socioeconomic environment.

**Trial registration:**

ID_RCB number: 2011-A00046-35

Clinicaltrials.gov number: NCT01425866

## Background

Type 2 diabetes (T2D) is a chronic and increasingly common disease linked to changes associated with sedentary lifestyles and inappropriate food energy intake [1]. The prevalence of known and treated diabetes is close to 4% in France, and up to 18% among adults aged over 30 years in Reunion Island, a French overseas region [2]. This situation gives rise to major costs, with overall expenditure in France estimated at €5,710 billion in 2000 [3]. In Reunion, the financial direct impact associated with diabetes and its complications is estimated at €5,451 per patient [4].

Intervention trials in T2D have clearly shown that complications can be prevented or delayed by rigorous control of the blood sugar level and risk factors [5,6]. An improvement of one point in HbA1c is associated with a 20% decrease in the occurrence of macrovascular complications and 30% to 40% decrease of microvascular complications [6]. Diabetes and the associated risk factors nevertheless remain poorly controlled for the most part [7,8]. In Reunion Island, as in France, glycaemic targets are not achieved: in the REDIA study, HbA1c was 8.4 ± 0.2% in patients treated with oral antidiabetic drugs (OAD) and 9 ± 0.5% in insulin-treated patients [2].

For subjects with T2D, four imperatives must be maintained over the long term to contribute to the prevention of complications: adherence to diet, physical activity, treatments and monitoring checks. In order to achieve these objectives the treatment, and optimum medical follow-up, must be associated with therapeutic patient education [9-11]. Self-management education (SME) aims to help patients to acquire or maintain the skills they need to manage their life with a chronic disease as effectively as possible [12]. These education actions must be based on solid theoretical foundations which take into account the complexity of the chronic disease [13-16] and the integration of the individual [15,17,18] in his or her social, family and cultural environment, as part of an appropriate approach taking into account social configurations [19,20] and capacities for change [21].

Several randomised trials assessing SME in T2D have been carried out in the last 20 years. Reviews and meta-analyses are available [22-25]. These have produced overall positive effects of intensive initial therapeutic education in T2D. However, results remain moderate beyond the short term (six months to 1 year). In known T2D, five to 10 years on average after diagnosis, improvements have been demonstrated in metabolic control, self-efficacy, self-management practices, satisfaction and quality of life [25], including in elderly subjects and ethnic minorities [26]. The currently available data underline the need to test structured and replicable group approaches with long-term educational multidisciplinary support, on precise theoretical bases and adapted to different publics and cultures [22,27,28]. Interventions calling for group education have two major attractions: reduced costs due to the grouping of several patients with a trainer, and effective learning time which enables patients to gain knowledge and to develop new skills. Nevertheless, the practice of group education could be difficult to implement, essentially because of organisational problems.

Whereas the effects of initial structured education have by and large been demonstrated at one year, the results remain moderate and appear to decrease thereafter. In a population of disadvantaged patients with poorly controlled diabetes, Brown et al., [29] showed the positive effects at one year of more numerous and repeated group sessions. The total effective education time improves the overall results of self-management programmes up to one year [22,29-32], but total uncertainty remains with regard to the question of providing on-going support with group education beyond one year for patients who received a structured initial education cycle [23-25,33]. The four-year ROMEO Italian trial [32] compared “systemic” group education with medical follow-up alone. The sessions were conducted on an outpatient basis within the diabetes secondary care structure. They showed a very positive effect on the metabolic control, increasing over time. The intervention comprised one hour education sessions delivered on a three monthly basis. The DESMOND programme, including a single initial 6-hr structured education cycle of six hours, in a recently diagnosed T2D, has had a favourable impact on weight, risk factors, beliefs and health practices, but no effect on the level of HbA1c at one and three years compared to a randomised control group [34,35]. A self-management intervention involving 10 two-hour group sessions in a primary care setting in Sweden, over a period of nine months, showed a 1.4% difference in HbA1c level at five years [36]. The requirement for long term maintenance closer to patients, at the community level and/or led by peer educators, has been stressed [37,38]. The expert patient education (X-PERT) programme delivered over six 2 hour-sessions reported HbA1c improvements at 14 months (−0.6% *v* 0.1%), although long term outcomes have not yet been reported [39].

In Reunion, the population remains socially and economically fragile on the whole [40]. A very high proportion −43.6% in 2002 – receive free complementary health cover. The REDIA-prev2 study conducted in 2004 in the two hospital centres in the north and the south of the island, failed to demonstrate the effect at one year of quarterly one-to-one counselling encounter with a registered nurse educator and a dietician [31]. Since 2004, the RéuCARE (Réunion Cœur Artères Rein Education) regional network in Reunion is based around the establishment of group SMEs in accordance with the *learning nests* approach whose theoretical bases draw on socio-constructivism, empowerment and cognitive social theories, and integrate the heterogeneity of individuals, including literacy level heterogeneity [41]. The educational group sessions take place at close quarters, organised all over Reunion Island. Educators are health professionals trained to hold learning sessions, with the learning nests empowerment approach. The present ERMIES study challenges the hypothesis that in difficult-to-control T2D a feasible and reproducible group-based structured empowering SME, lasting for two years at the proximity level, helps to achieve better metabolic control, as evidenced by the level of HbA1c, compared to an initial education cycle only. The ERMIES programme includes in both control and intervention groups an initial cycle made up of a minimum of three 2 hr long sessions in an empowerment model. The programme in the intervention group involves supplementary 4-monthly empowerment 2 hr sessions over 2 years.

## Methods/Design

### Trial design

**Figure 1:**
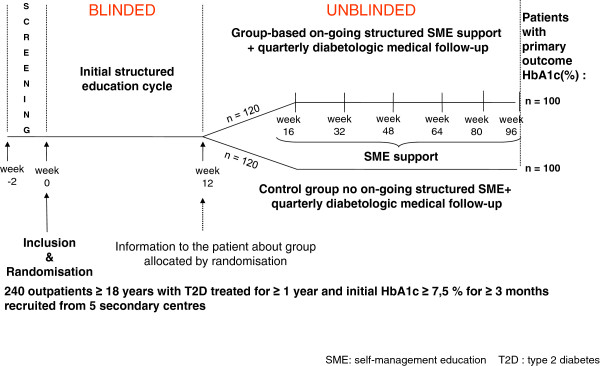
**Design of the protocol over two years in Reunion Island, ERMIES, 2011–2014** .

This is a multicentre open (except during the common initial education cycle) randomised two-arm controlled trial (Figure 1). This trial will be associated with a qualitative ethno-sociological study conducted on a sample of 30–40 persons.

Patients will be recruited from five different outpatient secondary settings in the four hospitals of the Reunion Island, and will be seen in outpatient consultations. Detailed information will be given by the investigating physician on the objectives of the study, its nature and constraints, the anticipated risks, and the expected benefits. Patients will be included in the study, and followed-up for two years, once a consent form has been signed.

### Eligibility criteria

1. Inclusion criteria: patients aged over 18 with type 2 diabetes, defined in accordance with WHO criteria treated for at least one year (OAD and/or insulin and/or GLP-1 analog); HbA1c ≥ 7.5% for three months or over; treatment regimen stable for at least three months (except for possible changes to insulin doses in the previous three months); living in Reunion Island, affiliated to French national health insurance, and patients having signed a consent form for participation in the trial.

2. Non-inclusion criteria: current or scheduled treatment liable to influence blood sugar levels, such as corticotherapy; current or recent hospitalisation (< three months) associated with the diabetes; structured education intervention in hospital or on outpatient basis in previous three months; severe evolving current complication: ischemic or proliferative retinopathy, severe chronic renal insufficiency (clearance < 15 ml/min), coronary heart disease, foot lesions; current pregnancy or desire for pregnancy during the trial; evolving cancer (except baso-cellular or spino-cellular cutaneous epithelioma); severe physical, cognitive or mental illness handicap; concomitant participation in a therapeutic intervention trial.

### Interventions

The patients randomised in both arms will benefit from a structured initial cycle. Trained educators, blind to the subsequent group allocation, will conduct this initial group education cycle within the 12 weeks following inclusion. At the end of these 12 weeks the blinding will be lifted, and the patients randomised in the two-year education arm will continue to receive on-going structured group education. All group sessions will take place at close quarters, organised by the RéuCARE health network in specific locations in each town (municipal and/or community settings).

Patients are reminded by telephone during the week prior to each educational session to confirm the date, time and place. Patients who fail to attend the session will be called within two days and offered a new schedule.

### Structured group SME intervention

1. The theoretical basis and framework of the *Learning nests*’ approach for the structured educational intervention [14,42].

The complex nature of chronic diseases requires that therapeutic education takes into account both its biomedical and psychosocial dimensions. Learning nests work on knowledge to be built, and social, individual and contextual dimensions of learning, as well as the required decision of actions on a long-term basis, taking into account the heterogeneity of individuals and of their conditions. The learning nests are based on the contributions of socio-constructivism [43,44], and empowerment [45], and take into account the enhancement of self-efficacy [18], through skill mastery, problem solving, decision making and action planning.

Fine components structure the approach of learning nest group sessions:

Component 1: Self analysis of relevant knowledge. The learner is placed in a condition to be able to analyse the relevant knowledge before making the decisions, which will be his own and not subordinated to those of the educator. Each learning nest offers a constructive meeting between the learners' ideas and the reference knowledge. Since the Learning nests do not include lectures, this meeting is dealt with through diversified actions exercised by the learner, on forms of knowledge that are made manifest and manipulable.

Component 2: Acknowledgement of individual contexts and problem-solving. The patient's "ordinary" context is legitimised by virtue of the visibility conferred on it (photographs, handouts, material, foods, ...). The learner can thus identify the affordances of his or her environment. The learner can continue to "manipulate" the reference knowledge in his own particular context. The encounter with the reference knowledge takes place in the presence of the health professional, and this body of knowledge is conveyed into the patients' context in the form of booklets that make sense to them, because the information has been put together there and then.

Component 3: Individual heterogeneity as an asset for self-assessment and action. The heterogeneity of the patients is not considered a problem, but as something of value, which is taken into account in the very act of designing the situation. The analytic dimension of the work to be carried out by patients is of importance. The specific individual characteristics of the patients can be integrated without exception. The outcome of each learning nest thus varies over a range of different solutions, and not a single one.

Component 4: The long lasting dimension of self management. The learning nests integrate the lasting dimension of chronic illness and the necessity of thinking the action over for a long time before seeing the first results. The decision for action always remains the property of the patient, with a space for encouraged actions in parallel, which leads the trainer to work with the patient's individual dispositions, helping the patient to become aware of the advantages and constraints of his environment. At the end of each group session patients identify a goal and an action plan related to their context. At the beginning of the each subsequent session patients are invited to reflect on their experience of working on their self-selected goals, and talk about what they had learned and how it could be applied in the future. Patients are encouraged to discuss events that had occurred and to reflect on how these events had affected self-management and action.

Component 5: Culturally tailored education materials. In addition to ensuring cultural relevance by focusing on the priorities and the personal contexts of the patients in the program, learning nests use education materials that are developed with and for the target audience: local foods, contextual photographs, natural surroundings, occupations, clothing, appropriate reading level, coloured items (for identification).

Learning nests incorporate and emphasise behavioural strategies, rather than a traditional didactic approach, including curricula and handouts that have been developed to meet the specific needs of the target population [14,19,40,46]. They also involve psychosocial dimensions, and the presence of the context in which the chronic illness is dealt [47] with on a daily basis, and the patient’s need for a decision on actions to be taken.

The learning nest can be defined as a learning empowerment situation centred on the construction of bodies of knowledge, in which the knowledge worked on in a given situation will enable the patient to take decisions relating to his health management. The patient has the possibility to modify his everyday practices (nutrition, physical activity, taking medication) while bearing in mind his/her existence in relation with the others, with his environment, his resources and his culture. Patients become aware of the issues they can manage on a priority basis, taking account of not only of medical dimensions (level of HbA1c, other risk factors, treatment, etc.), but also psycho-sociological, contextual, environmental, cultural and economic dimensions. This work results in a decision which incorporates a positive view of health and feasibility in contexts in which the patient will develop. Whatever the specific cultural, religious and socio-economic background of the patients, each can work on the basis of his own data, reinforce his own views and find a way ahead which takes into account the constraints and strengths of his social, family and personal life.

The material, process and the design of each educational group session are detailed in specific booklets for learners, including those with literacy difficulties. Booklets for educators include a curriculum written protocol, available in French and in the English language (EPMC booklets; *Education Prévention des Maladies Chroniques*[48]), allowing replication of the educational intervention.

2. Initial education cycle

The initial cycle consists of 3 to 7 educational weekly or bi-weekly 2-hour group sessions for 4 to 10 patients:

Personalised assessment group session (120 minutes): patients work on their own clinical and biological factors that can influence health maintenance (blood glucose, blood pressure, lipids, weight, waist circumference, smoking), before looking at lifestyle measure effects and specific impacts of eventual changes. They then scrutinise the feasibility of action planning and implementation in real life, and goal-setting taking into account all individual, social, and contextual dimensions. At the end of this session, each learner can schedule 2 to 6 specific additional focused sessions, according his/her needs and goal-setting.

Two to 6 specific additional focused sessions of 90 to 120 minutes, spaced at intervals of 1 to 2 weeks (3 weeks maximum). These sessions are selected on the six following themes: self-monitoring and adaptation of treatment - physical activity - food and control of fat intake – understanding of diabetes and treatment – challenges of insulin therapy – prevention of foot lesions.

3. On-going SME support: personalised empowerment follow-up including group sessions of 90 to 120 minutes each, with three to ten patients at 16, 32, 48, 64, 80 and 96 weeks. Each session will be scheduled at the end of the previous session. Patients will work on the progress of their indicators as follows: medical (HbA1c levels, blood sugar, arterial pressure, lipids, waist circumference, smoking, treatments); health coping and goal setting (action(s) initially decided on, actual implementation, obstacles); cognitive (review of knowledge gained through the first session assessment session and additional sessions). The summary of the session comprises objectives for action(s) to be implemented and the scheduling of optional additional sessions to support practical implementation.

4. Educators

The professionals carrying out the sessions have been specifically trained (long-term training by the University Diploma on SME in chronic diseases, or training with experience and skills validated in the network). Training of educators include sessions on biomedical knowledge on diabetes, nutrition and treatment, educational skills, behavioural strategies, learning nest and empowerment approach. In order to “embrace empowerment”, health care professionals have to make a paradigm shift from the traditional approach to care and acknowledge that patient are in control of their daily diabetes care, making autonomous and informed decisions about their diabetes self-management [49]. Additionally, training includes for each thematic group sessions a thorough review of the process, content, educator role, learners actions, learning indicators, specificity of goal setting and problem solving taking into account individual, social and cultural context.

### Control arm: group-based initial structured education cycle only

In the control arm, patients receive only the initial structured education programme, conducted in the 12-week period following inclusion.

### Concomitant treatments and procedures

In both arms, patients will continue to receive quarterly outpatient medical follow-up. Regular examinations and therapeutic adaptation will take place in accordance with the recommendations. The individual nursing or dietician encounters, required in addition to the medical appointments due to the evolution of the disease or therapeutic changes (such as starting insulin, GLP-1 analogue) will be authorised whatever the arm.

### Outcomes

The main outcome is the evolution of HbA1c (%) at two years. The HbA1c will be measured by the HPLC method in a centralised laboratory (biochemistry laboratory of the Félix Guyon hospital in St Denis).

The secondary outcomes will be as follows: quarterly evolution of HbA1c (%), fasting blood glucose, anthropometric indicators (body mass index, waist circumference), blood pressure, tobacco consumption, annual screening of lipids (total cholesterol, triglycerides, HDL and LDL cholesterol), antidiabetic treatments (type and dose) and the occurrence or worsening of diabetic complications. Health practices (level of physical activity and food consumption) will be assessed by means of annual questionnaires. These have been used in Reunion Island for a number of descriptive or intervention studies: RECONSAL [50], REDIA-prev1 [51] and REDIA-prev2 [31]:

Physical activity: physical activity scores will be assessed by means of a questionnaire developed on the basis of the Baecke self-questionnaire [52], which explores professional, sporting and leisure exercise. Questions on domestic activity were added to our studies.

Food consumption: food consumption (reported energy intake, macronutrient intake, dietary habits) will be assessed by means of a rapid food frequency questionnaire relating to weekly consumption [53]. Quantities of various foods are assessed by means of a photo album. The validity of the questionnaire and the photo album for the population of Reunion was checked by comparison with food surveys.

The care and treatment courses will be assessed by means of questionnaires at inclusion and at two years, based on the questionnaire used in the French ENTRED study [54] adapted to the situation and context in Reunion and comprising 30 items.

Quality of life will be assessed annually by means of the Diabetes Quality of Life, Brief Clinical Inventory [55]. This scale, including 15 items and translated into French for Reunion, was validated for this trial among a sample of 150 Reunion’s patients presenting diabetes.

Self-efficacy, social support and anxio-depressive state will be assessed by means of psychometric scales validated for the purpose of the trial in Reunion. The feeling of self-efficacy will be measured by means of section 3 of the MDQ (Multidimensional Diabetes Questionnaire) [56]. This questionnaire includes 13 items: seven for self-efficacy and six for outcome expectations. The patent’s perception of the specific social support from the partner with regard to the treatment of the diabetes will be assessed on the basis of 12 items derived from the MDQ. The patient’s anxiety will be assessed by the HADS (Hospital Anxiety and Depression Scale) [57]. The HADS includes seven items and is widely used and validated in outpatient prospective studies. The depression level will be measured by means of the CES-D [58,59], which comprises 20 items. The French versions of the two latter tools have demonstrated their psychometric qualities.

The food consumption questionnaire will be conducted face to face with the centre nurse trained for this purpose before the start of the research. The other questionnaires will be self-administered, with the assistance of the study nurse if necessary for subjects with low literacy levels.

### Sample size consideration

The efficacy assessment is based on the following main judgement criterion: developments between inclusion (week 0) and two years (week 96) of the percentage of glycated haemoglobin HbA1c (%).

The expected benefit is a decrease at two years of at least 1% in the average HbA1c between the group “structured on-going SME support” and the control group “Initial structured education cycle only”. An absolute difference of 1% at two years between the two groups appears relevant [23,24,29,32,39,60-62].

To show an absolute difference of 1% of HbA1c, with a standard deviation of 2.5%, a type I error rate of 5% and a power of 80%, it is necessary to include 99 subjects per group in a bilateral situation.

Because of an estimated data deficiency of 20% at two years (drop out, refusals, deaths), it is planned to include 120 subjects per group, making a total of 240.

### Randomisation methods

Block randomisation is carried out on two stratification factors: insulin treatment on inclusion (yes/no - two strata), and inclusion centre (five strata). The numbers of participants in each group are balanced with a ratio of 1:1. The 10 randomisation lists are drawn up by the statistician of the CIC-EC of Reunion before the start of the research.

### Blinding

For all patients, the first phase (group-based initial education cycle) will be conducted blind with regard to the randomisation arm for patients, until 12 weeks at the latest, researchers and education team. At the end of this first phase the follow-up will therefore be unblinded.

### Statistical methods

The statistical analyses will be carried out at the CIC-EC in Reunion, under SAS® (version 9.2, SAS Institute Inc., Cary, NC, USA).

Description of the initial characteristics of the screened patients and of the included patients will be done by usual statistics (number, percentage, average ± standard deviation or median and range).

Effective implementation of the SME program and the effective participation will be documented.

The assessment of efficacy of the intervention will be performed in a single analysis, according to the intention to treat principles (*i.e.* according to group allocated by randomisation) with type I error equal 5%, and bilateral formulation of Student t-test (primary analysis).

In the two compared groups, the deficient data on HbA1c (%) at two years (week 96) will be replaced by the measurement obtained at week 84.

If the primary analysis shows a significant difference between the two groups, a comparison of the mean change in HbA1c (%) S96-S0 will be done by the level of participation (secondary analysis using 1-factor ANOVA model): i) basic level ("initial structured therapeutic education only"), ii) level 1 (“structured on-going SME support” without specific additional focused sessions), iii) level 2 (“structured on-going SME support” with specific additional focused sessions).

Analysis of longitudinal data gathered during two years will used in generalised estimating equations (GEE) adapted to repeated measurements in order to test the trends described over the duration of the supervision.

Analysis of the time between randomisation and occurrence of a level of HbA1c <7% (first event observed) will be performed by the Kaplan-Meier method, with comparison of survival curves between groups by Log-rank test.

At each annual collection point the anti-diabetic treatments will be classified into four hierarchical categories: 1) non-insulin antidiabetic monotherapy, 2) non-insulin antidiabetic bi-, tri- (or more) therapy, 3) one injection of insulin, 4) two or more injections of insulin. Between two collection points, moves from one category to a higher (or lower) category will be identified as a reinforcement (or reduction) of the treatment. The interaction between the intervention and treatment category will be tested in the appropriate multivariate models (ANCOVA, GEE, time-dependent variable in the Cox model).

### Nested qualitative study

The associated qualitative study will be conducted on a sample of 30 to 40 persons in both arms, and will include observation and recording group sessions as well as semi-directive conducted interviews at home.

The objectives of the ethno-sociological qualitative study are: i) to analyse the learning processes during the education sessions, and then in individual contexts to analyse the developments in health ‘literacy’ is taken to mean not only reading and writing ability and numeracy, but also information searching, cognitive work and decision-making [63] in the various areas covered by the SME process. These areas are: food, physical activity, control of risk factors in complications, blood sugar self-monitoring and adaptation of the treatment. 2) analysing the adaptation processes in context and their links with the sessions, interactions in the education session and literacy levels; 3) endeavouring to generate barriers and enablers in developing self-management.

The participants in the qualitative study will be selected on a non-random basis by the head of the ethno-sociological survey (M. Balcou-Debussche), so as to have a sufficiently wide panel of participants, taking account of the age, sex, length of time since the onset of diabetes and its level of control and complications, professional activity, habitat, socio-economic level and level of education. The possibility of inclusion in the ethno-sociological research (including interviews at home) will be specified in the information letter and consent form. Additional oral information will be provided for a qualitative study of participants on the aims and precise conditions of this survey.

An analysis will be made of the content of the themes covered in the interviews and the recurring themes. The categories and signifiers will be developed using the constant comparison method. The emerging themes can be included and developed in new analysis and data-gathering cycles [64]. The session observations will include an ethnographic summary of language interactions, speaking and activities of participants. All the data, themes and recurrence will be the subject of cross-analyses of the same individuals according to the context, sessions or interviews. The analyses will also cover the evolution since the beginning of the research, as well as differences between individuals. All the data will be coded and analysed with the aid of the N-Vivo 9 software QSR international.

### Collection of biological samples

Serum collection will be performed at inclusion, as well as at one year and two years for the purpose of a posteriori secondary analyses on the evolution of more specific indicators of the risk of long-term complications of type 2 diabetes. In particular, inflammation and immunity factors, oxidative status and lipid fractions (size and density of LDL, HDL fractions, atherogenic lipoproteins).

### Ethics committee

This study has received approval from the ethics committee, “CPP Sud-ouest et Outre Mer III” and authorisation from the French Health Products Safety Agency (*Agence française de sécurité sanitaire des produits de santé*, Afssaps).

## Discussion

Available studies which were carried out on the basis of these inclusion criteria (adult diabetes patients remaining at very high risk of complications due to persistent poor blood glucose control with HbA1c in excess of 7.5%) have shown a favourable effect from educational intervention at six months and one year, but the question of the efficacy of maintenance at two years, and of intensification by means of on-going structured intervention at the proximity level, still remains unanswered as compared to a sole initial structured education.

The relatively heterogeneousness of these patients showing insufficient therapeutic control (treatment by OADs or GLP-1 analogue, basal or multi-day insulin therapy) may give rise to significant differences in metabolic control. Randomisation with stratification into two groups in accordance with the addition of insulin therapy will make it possible to balance the spread of treatments between the two arms at the time of inclusion, and to assess situations presenting comparable initial medication treatments. On the other hand, anti-diabetic treatments may mask the effects of therapeutic education on the level of blood glucose control: i.e. the drug therapy reinforcement required due to the lower efficacy of the education in one group could minimise the difference between the two intervention groups. Although that is not the case in the available studies that have assessed this issue, it appears important to record the changes of anti-diabetic treatments occurring throughout the study [22,23,39].

Maintained structured educational on-going support over two years requires co-ordination, in parallel with medical and diabetological supervision, thanks to the existence of an education coordination network in Reunion. The network has the resources and already conducts structured education cycles. Self management education sessions are carried out by trained and experienced health care professionals, with an empowerment approach. The SME style is theoretically based and reproducible, with curricula and handouts making it possible to renew educational actions on the same bases. The potential investment of peer educators or expert patients has not been retained for this ERMIES study design, since the French and Reunion context is currently not yet ready to include such issues. However, this is an important clue to evaluate and implement in the future [37-39].

The primary outcome will be based on the evolution of HbA1c at two years, in order to measure the maintenance of improved metabolic control by means of a reliable and representative gauge of the long-term risk of microvascular and macrovascular complications. The purpose of educational intervention on an individual and on a collective level is to prevent the occurrence of secondary complications in the long term. Demonstrating a significant effect on the prevention of secondary complications will nevertheless take several years [5,6], but HbA1c and cardiovascular risk factors are solid intermediate outcomes in the case of type 2 diabetes. Since it appears difficult to envisage a combined outcome of the various cardiovascular risk factors with HbA1c, we have chosen to retain HbA1c as the main outcome, particularly since most of the available studies have been based on it.

The qualitative, ethno-sociological study [14] will allow a more detailed analysis of the determinants and processes enabling people to make progress in the control of the disease, taking into account the interactions during the session, but also particular contexts, (individual, social, cultural factors) [65], and potential strengths to be leveraged for improved health literacy, which is deemed to mean the integration of significant processes to acquire information and knowledge, understanding and decisions [63].

The efficacy and potential significance of organising education groups at close quarters over the long term are important to specify. Delivering to each patient an individualised multidisciplinary therapeutic education requires time and human resources, and entails a significant cost. On the other hand self-management group education is difficult to structure, and requires organisational capacities of teams and professionals, but it ultimately enables the individual approach to be reserved for cases that genuinely need it. Furthermore, the modalities of patient-adapted group education are still being debated. The results of the present study will thus indicate whether such long-term structuring patient education in insufficiently controlled diabetes provides a real benefit, as compared to initial education over several weeks. The possibility, within the fabric of the current healthcare system, particularly in France and Reunion, of offering structured, community-based education to patients in difficulty opens up the potential to provide support for specialist and hospital teams which are facing increasing demand for type 2 diabetes management. The link to a qualitative ethno-sociological study will provide knowledge of the learning processes in context, and change factors in practices, which will make it possible to adapt and direct future actions in terms of prevention and therapeutic education.

## Abbreviations

EPMC: Education Prévention des Maladies Chroniques; ERMIES: Multicentre randomised trial of structured educational intervention in poorly controlled type 2 diabetes; OAD: Oral antidiabetic drugs; SME: Self-management education; T2D: Type 2 diabetes.

## Competing interests

The authors declare that they have no competing interests.

## Authors' contributions

XD was the initiator for this study, XD, FC and AF drafted the study protocol. XD organised the recruitment of the centres. XD, FF, AF, and MK developed the questionnaires, FC, AF, FF organised the data collection and management and the administration of the whole study. MBD and XD developed the SME programme; MBD drafted the protocol of the nested qualitative study. AF conducted the power calculation, supervised the analyses and gave statistical and methodological input. IFR and XD are the organisers of the nurses' education course and patient self-management group education follow-up. XD, FC and AF wrote and revised the final manuscript, and all authors read and approved it.

## Funding

Programme Hospitalier de Recherche Clinique interrégional, Ministry of Health, France, 2010.
